# eEF-2 Phosphorylation Down-Regulates P-Glycoprotein Over-Expression in Rat Brain Microvessel Endothelial Cells

**DOI:** 10.1371/journal.pone.0125389

**Published:** 2015-05-11

**Authors:** Xing Hua Tang, Xun Yi Wu, Lan Xu, You Xin Fang, Ping Wang, Guo Xing Zhu, Zhen Hong

**Affiliations:** Department of Neurology, Huashan Hospital, Fudan University, Shanghai, China; Massachusetts General Hospital/Harvard Medical School, UNITED STATES

## Abstract

**Objective:**

We investigated whether glutamate, NMDA receptors, and eukaryote elongation factor-2 kinase (eEF-2K)/eEF-2 regulate P-glycoprotein expression, and the effects of the eEF-2K inhibitor NH125 on the expression of P-glycoprotein in rat brain microvessel endothelial cells (RBMECs).

**Methods:**

Cortex was obtained from newborn Wistar rat brains. After surface vessels and meninges were removed, the pellet containing microvessels was resuspended and incubated at 37°C in culture medium. Cell viability was assessed by the MTT assay. RBMECs were identified by immunohistochemistry with anti-vWF. P-glycoprotein, phospho-eEF-2, and eEF-2 expression were determined by western blot analysis. *Mdr1a* gene expression was analyzed by RT-PCR.

**Results:**

*Mdr1*a mRNA, P-glycoprotein and phospho-eEF-2 expression increased in L-glutamate stimulated RBMECs. P-glycoprotein and phospho-eEF-2 expression were down-regulated after NH125 treatment in L-glutamate stimulated RBMECs.

**Conclusions:**

eEF-2K/eEF-2 should have played an important role in the regulation of P-glycoprotein expression in RBMECs. eEF-2K inhibitor NH125 could serve as an efficacious anti-multidrug resistant agent.

## Introduction

Multidrug resistance represents a serious obstacle to achieving a successful seizure-free status in epilepsy. Although multifactorial in etiology, one type of multidrug resistance is associated with the over-expression of energy-dependent membrane-bound pumps, which intercept and efflux drugs before they reach their intracellular target structures. P-glycoprotein (P-gp, MDR1) represents a paradigm ATP-dependent efflux pump and is physiologically expressed in a number of tissues including endothelial cells that form the blood-brain barrier (BBB) [1]. P-glycoprotein may alter the distribution and elimination of its substrates[2,3]. When over-expressed, P-glycoprotein may confer multidrug resistance, leading to failure of drug therapies for epilepsy[4] and cancer[5].

Studies have shown that glutamate could induce P-gp over-expression via N-methyl D-aspartate (NMDA) receptors in rat brain microvessel endothelial cells (RBMECs)[6,7]. Excitotoxicity induced by glutamate involves an intracellular increase of Ca^2+^through excessive stimulation of NMDA receptors[8]. Ca^2+^ plays an important role in activating several kinases as secondary signaling messengers. Studies on protein translation in non-neuronal systems have indicated a role for Ca^2+^/calmodulin in the inhibition of polypeptide elongation, which results from the phosphorylation/dephosphorylation of eukaryote elongation factor-2 kinase (eEF-2K) (2, 4, 5) and eukaryotic elongation factor-2 (eEF-2)[9,10]. eEF-2K, which is also known as Ca^2+^/calmodulin–dependent kinase III, was first identified by Nairn et al.[11]; it regulates many cellular processes through its essential role in protein translation[12,13]. eEF-2 kinase controls the movement of the elongating peptide chain by specifically phosphorylating eEF-2 at Thr56[14,15], which decreases the affinity of the elongation factor and cognate peptide for the ribosome[12,16]. eEF-2 is the sole substrate of eEF-2K; it does not appear to be phosphorylated by any other kinase[17,18].

The aim of the present study was to confirm the glutamate-NMDA receptor-Ca^2+^/calmodulin-eEF-2K/eEF-2 P-gp signaling pathway and measure the effects of the NMDA antagonist MK-801 and the eEF-2K inhibitor NH125 on P-gp expression. MK-801 is a noncompetitive antagonist that binds to the ion channel of NMDA receptors, producing an almost irreversible blockade, including of calcium influx[19]. NH125 was first reported as a potent agent against drug-resistant bacteria through inhibition of histidine protein kinase[20–22]. Later, it was found that NH125 was indeed a potent and selective inhibitor of mammalian eEF-2K in vitro[23]. Additional experiments demonstrated that NH125 was efficacious against a broad spectrum of human cancer cell lines in vitro and in vivo [23,24]. These findings suggested that NH125-mediated anticancer activity was due to inhibition of eEF-2K. Therefore, NH125 can be used as an eEF-2K inhibitor to observe the effects on the eEF-2K/eEF-2 pathway.

**Fig 1:**
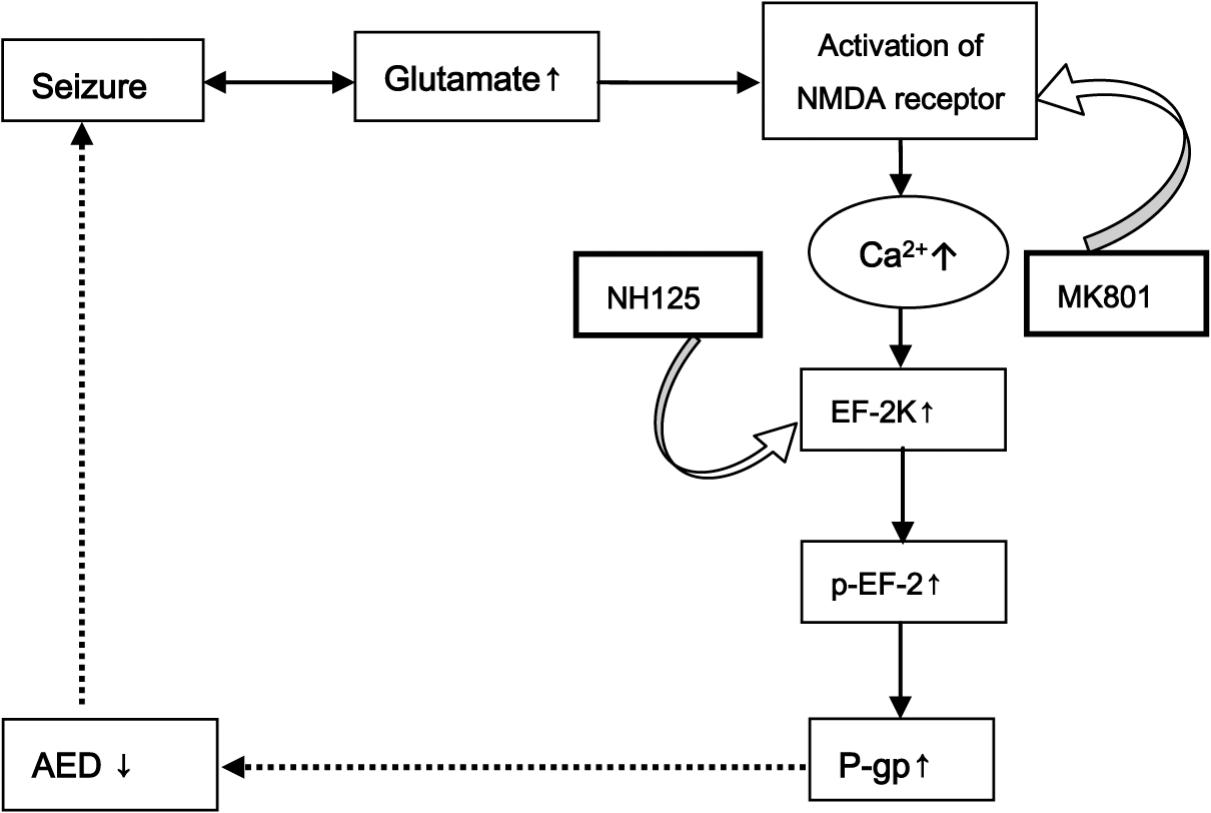
Schematic of proposed glutamate-NMDA receptor-P-gp signaling pathways and possible intervention sites.

Here, we investigated the glutamate-NMDA receptor-Ca^2+^/calmodulin-eEF-2K/eEF-2 P-gp signaling pathway and looked for the key modulators on NMDA receptors or eEF-2K/eEF-2 that regulate P-gp expression in glutamate-induced up-regulation of P-gp expression in RBMECs. We observed the effects of the NMDA antagonist MK-801 and eEF-2K inhibitor NH125 on P-gp expression. The expected aim of our study were shown in Fig 1.

## Materials and Methods

### Materials

P-gp, eEF-2K, phospho-eEF-2 (Thr56) monoclonal antibody was purchased from Abcam Chemical Co. (Cambridge, UK). Fluorescein isothiocyanate (FITC)-labeled rabbit anti-rat IgG was purchased from Jackson Immunoresearch (West Grove, PA, USA). NH125, L-glutamate, MK-801, N-acetylcysteine and anti-vWF were obtained from Sigma Chemical Co. (St. Louis, MO, USA). All other chemicals were of analytical grade and are commercially available.

### Ethics Statement

All experiments were carried out in accordance with guidelines evaluated and approved by the ethics committee of Fudan University, and the experimental animals use license was SYXK (Shanghai) 2009–0082. The housing facility of experimental animalswas a barrier housing facility, and it has kept with national standard’ Laboratory Animal—Requirements of Environment and Housing Facilities’(GB 14925–2001). The care of laboratory animal and the animal experimental operation have conforming to’Shanghai Administration Rule of Laboratory Animal’. All efforts were made to minimize pain or discomfort in the animals used. The rats were sacrificed with dislocation when anaesthetized under -20°C.

### Animals

We got newborn 1-day-old Wistar rats from Department of Laboratory Animal Science, Fudan University, Shanghai, China. All studies were carried out in the laboratory of the Institute of Neurology, Huashan Hospital, Fudan University, Shanghai, China.

### Isolation and culture of primary rat brain microvessel endothelial cells (RBMECs)

Endothelial cells were isolated from rat brain according to a modified version of a method described previously[25]. Briefly, cortex was obtained from newborn, 1-day-old Wistar rat brains and placed in ice-cold phosphate-buffered saline (PBS). After removing surface vessels and meninges, cortex gray matter was minced and incubated at 37°C for 20 min in D’hanks solution. Then the samples were passed through a 150μm nylon mesh. After centrifugation at 2000 rpm for 5 min, the pellets was resuspended in PBS containing 25% bovine serum albumin (BSA) and centrifuged at 2000 *g* at 4°C for 10 min. Fat, cell debris, and myelin floating on the BSA were discarded and the pellet containing microvessels was resuspended and incubated at 37°C for 30 min in Dulbecco’s modified Eagle medium (DMEM) containing 0.1% collagenase II (containing Dnase I, 30U/mL). The microvessels were finally collected by centrifugation at 2000 rpm for 5min; then the pellet was washed twice with PBS and maintained in culture medium consisting of DMEM supplemented with 20% heated-inactivated fetal bovine serum, 1ng/mL bFGF, 100 kU/L penicillin and 100 mg/L streptomycin in culture dishes precoated with gelatin.

### Identification of primary RBMECs

After 7–8 days of culture, we observed and calculated the density of primary RBMECs by an image analysis system (Image-Pro Plus, Media Cybernetics Incorporation, USA). When the growth density of RBMECs reached 1*10^3^/mm^2^,they were postfixed with 4% paraformaldehyde for 20 min at 4°C and then washed extensively in PBS (pH 7.4). Following blockade of endogenous peroxidase activity by incubation with 0.5% H_2_O_2_ for 20 min, RBMECs were again washed with PBS and incubated with blocking solution (3% BSA, Sigma-Aldrich; 0.3% Triton X-100 in PBS) at room temperature for 20 min. Thereafter, RBMECs were incubated overnight at 4°C with a monoclonal rabbit anti-vWF antibody (1:200). After RBMECs were washed with PBS, they were incubated with secondary antibody (red fluorescein rabbit anti-rabbit antibody, dilution 1:1000; Jackson Immunoresearch, PA, USA) for 60 min. RBMECs were washed again with PBS, air-dried, and placed on cover slips precoated with glycerol.

### Cell viability assay

After primary RBMECs were cultured for 7 days, RBMECs in 96-well plates were exposed to L-glutamate (10–3000 μM) or NH125 (10–1000 μM) for 30 min at 37°C in 5% CO_2_. The L-glutamate and NH125 were dissolved in PBS solution supplemented with DMEM. After the treatments, the solutions were replaced with DMEM culture medium at 37°C for 24 h, after which cell viability was assessed using the MTT assay. MTT (0.5g/L) was added to the medium, and after an additional 4 h incubation, the medium was aspirated and the formazan crystals were dissolved in 200 μL DMSO. The cell viability of cultures treated with L-glutamate or NH125 was determined according to OD values at 570 nm measured by a microplate reader. The cell viability = OD value of treated group /OD value of control group * 100%.

### Treatment with L-glutamate/MK-801 and NH125

After primary RBMECs were cultured for 7 days, cells were divided into four groups: MK-801 + glutamate group (MK-801 + Glu), PBS + glutamate group (Glu + PBS), NH125 + glutamate (NH125 + Glu) and a control group. For the MK-801 + Glu group, 100μM glutamate was added to the culture medium for 30 min. The NMDA antagonist MK-801 (100 μM) was present for the pre-incubation time of 15 min as well as during glutamate exposure. For the NH125 + Glu group, 100 μM glutamate was added to the culture medium for 30 min. The eEF-2K antagonist NH125 (100 μM) was present for the pre-incubation time of 15 min as well as during glutamate exposure. After incubation, the medium was discarded and the cells were washed twice with PBS and the medium was appended to each well. After an additional 1, 3, 6, 24, or 72 h incubation, the medium was discarded and cells were washed three times with cold PBS, before being collected. For the Glu + PBS group, we replaced the MK-801 with PBS. For the control group, we replaced MK-801 and L-glutamate with PBS.

### RT-PCR analysis of *mdr1a* gene expression

Total cellular RNA was isolated from treated cells or control samples using Trizol reagent. RT-PCR analysis of *mdr1a* and *β-actin* expression was performed according to a modified protocol[26]. cDNA prepared from 20 μg of total cellular RNA was used for PCR amplification with specific primers for rat *mdr1a* (sense primer: 5′-TTTCAAAGGTTGTAGGGG-3′; antisense primer: 5′-CAATGTATCGGAGTCGC-3′, 180 bp) and the control *β-actin* (sense primer: 5′-AACCCTAAGGCCAACCGTGAAAAG-3′; antisense primer: 5′-TCATGAGGTAGTCTGTCAGGT 3′, 241 bp). PCR amplification of cDNA was run at 95°C for 30 s, 56°C for 30 s, and 72°C for 40 s for 40 cycles (under our experimental conditions, 40 cycles was optimal for the quantification of *mdr1a* mRNA expression). The RT-PCR products were visualized by electrophoresis on 2% agarose gels containing 0.5 μg/mL ethidium bromide. Analysis of the RT-PCR products was conducted using scanning densitometry.

### Western blot analysis

The cells were lysed in 125 mMTris-HCl (pH 6.8), 2% SDS, 10% glycerin, 20 mM dithiothreitol, 1 mM EDTA, and 0.01% bromophenol blue. Equal amounts of proteins (30 μL per lane) were separated by 6% SDS-polyacrylamide electrophoresis gels and transferred to nitrocellulose membranes. Nonspecific binding sites were blocked by a 1 h incubation of the nitrocellulose membranes in PBS containing 5% nonfat dried milk and 0.2% Tween 20 (blocking buffer), then immunoblotted with anti-P-gp, anti-eEF-2K, anti-phospho-eEF-2 (Thr56), and anti-β-actin antibodies. Detection by enzyme-linked chemiluminescence was carried out according to the manufacturer’s protocol. The membranes were washed in PBS, and bands were visualized using a Gel Image System (Tanon, Beijing, China) and western blotting analysis system.

### Statistical analysis

Each experimental step wasdone with four copies simultaneously, and all experiments were repeated at least twice. Some parts of experiments were did three times to confirm the results. Statistical analysis was performed with SPSS. Data are presented as means ±SE. Differences between groups were assessed by one-way ANOVA or one-way repeated-measures ANOVA where appropriate. The level of significance was set at *P* < 0.05.

## Results

### Culture and identification of primary RBMECs

**Fig 2:**
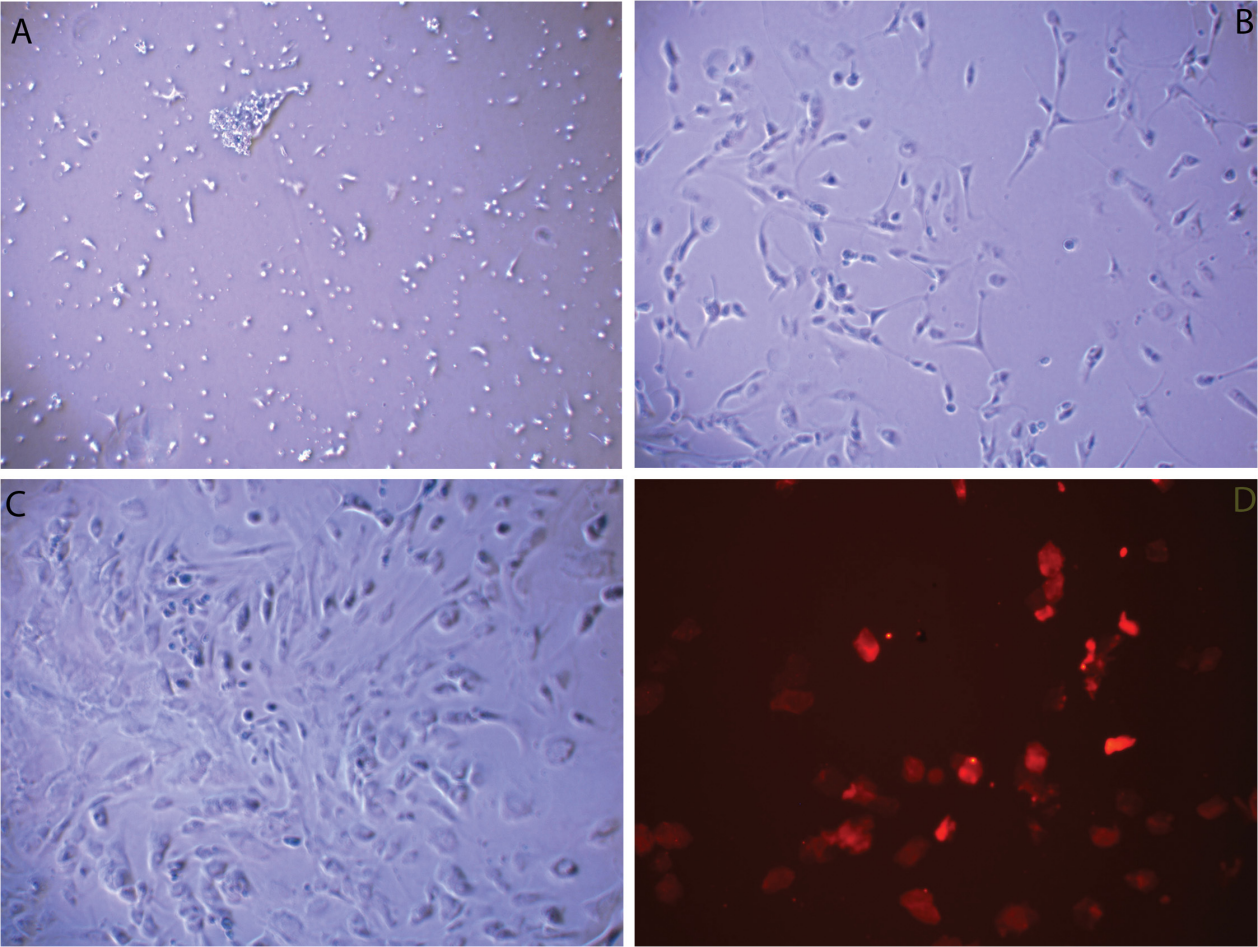
Culture and identification of primary RBMECs. (A) Circular RBMECs were seen at the moment of inoculation. (B) Four days after inoculation, adherent, coupled and proliferated growth of RBMECs was seen. (C) Seven days after inoculation, the RBMECs tended to fuse together. (D) To identified the primary RBMECs, we used anti-vWF (1:200) as a marker; the cytoplasm of primary RBMECs is wholly stained in red. To observe the RBMECs, cells were inspected at 100 × magnification.

Observed through a microscope, single-branch or branched brain capillaries that were composed of circular endothelial cells were seen at the moment of inoculation (Fig 2A). Three to four days after inoculation, adherent, coupled and proliferated growth of endothelial cells was seen (Fig 2B). As the incubation period became longer, short spindle endothelial cells cloned and clustered. Up until seven days, the cells tended to fuse together(Fig 2C). Seven days after inoculation, we identified the primary RBMECs with anti-vWF (1:200), as a marker of brain microvascular endothelial cells(Fig 2D; the cytoplasm of primary RBMECs is stained in red).

### Viability of primary RBMECs

#### The effect of L-glutamate on the viability of primary RBMECs

**Fig 3:**
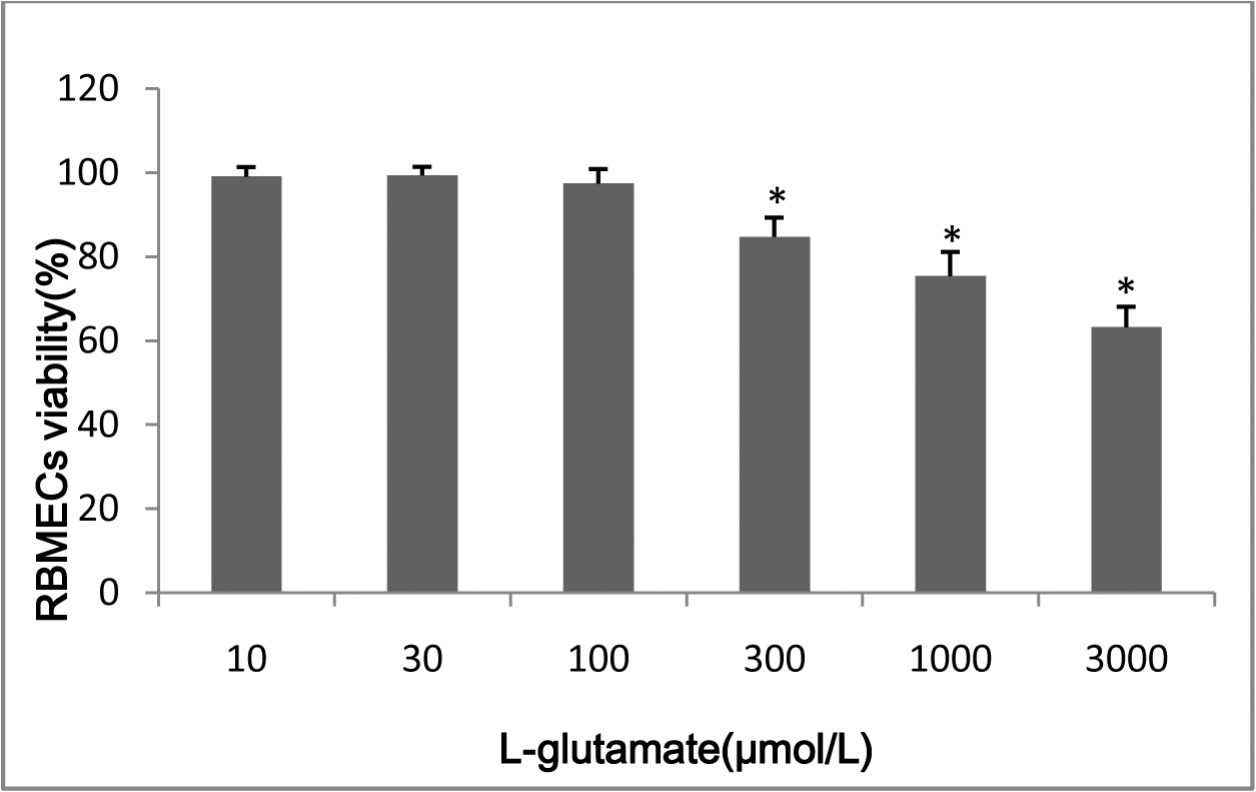
The effect of L-glutamate on the viability of RBMECs. The asterisk represents *P*<0.05.

The effect of L-glutamate on the viability of primary RBMECs, as determined by the MTT assay, is shown in Fig 3. After primary RBMECs were exposed to 10, 30, 100, 300, 1000, and 3000 μM glutamate for 30 min, followed by incubation for a further 24 h, the relative cell viability was 99.1%, 99.4%, 97.4%, 84.8%,75.4% and 63.2%, respectively. No obvious cytotoxic effect of L-glutamate at a concentration of 10, 30, or 100 μM was observed. Therefore, all further investigations on the effects of L-glutamate on primary RBMECs were restricted to 100 μM glutamate.

#### The effect of NH125 on the viability of primary RBMECs

**Fig 4:**
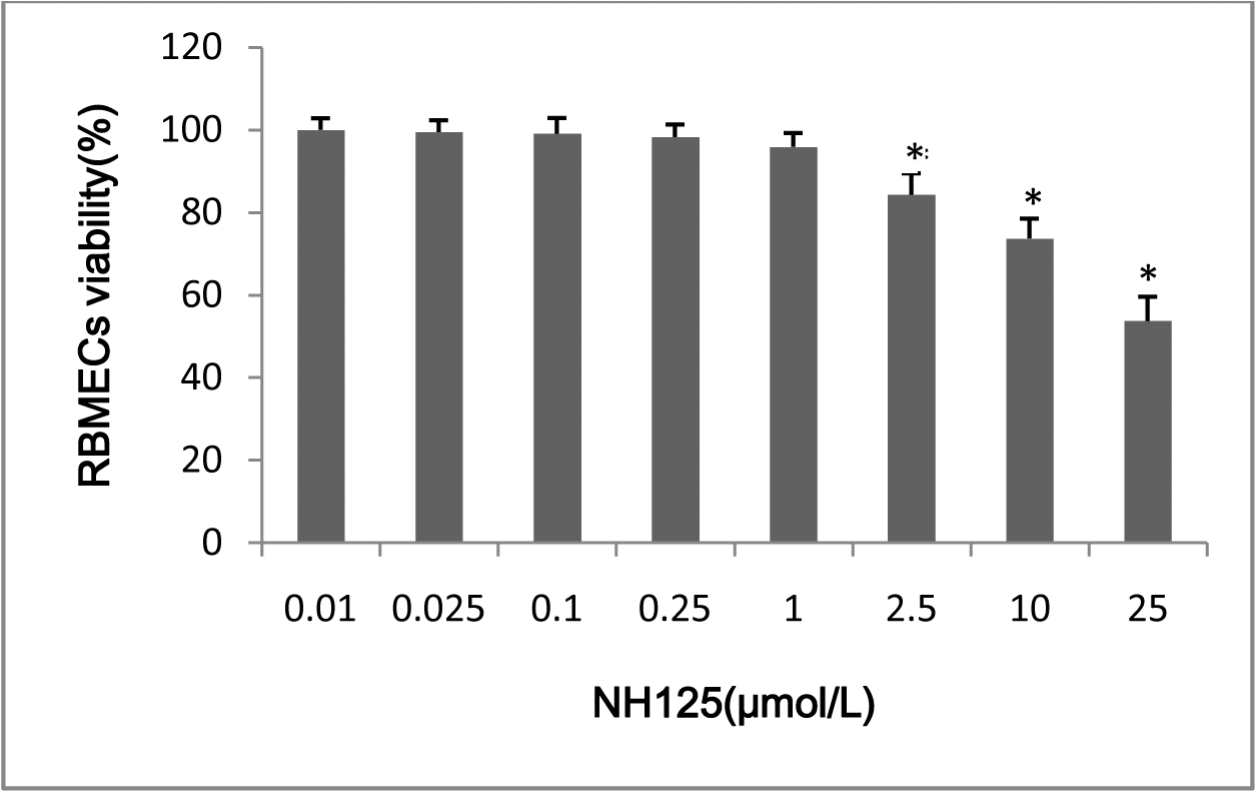
The effect of NH125 on the viability of RBMECs. The asterisk represents *P*<0.05.

The effect of NH125 on the viability of primary RBMECs is shown in Fig 4. After primary RBMECs were exposed to 0.01, 0.25, 0.1, 0.25, 1, 2.5, 10, and 25 μM NH125 for 30 min, followed by incubation for a further 24 h, the relative cell viability was 100.0%, 99.5%, 99.1%, 98.3%,95.9%, 84.3%, 73.7%,and 53.7%, respectively. No obvious cytotoxic effect of NH125 at a concentration of 0.01, 0.25, 0.1, 0.25, or 1 μM was observed. Therefore, all further investigations on the effects of NH125 on primary RBMECs used a concentration of 1 μM NH125.

### Effect of L-glutamate/MK-801 stimulation on P-gp/*mdr1a*/eEK-2K/phospho-eEF-2 expression

#### *Mdr1a* mRNA expression increased in L-glutamate-stimulated primary RBMECs, and was inhibited by MK-801

**Fig 5:**
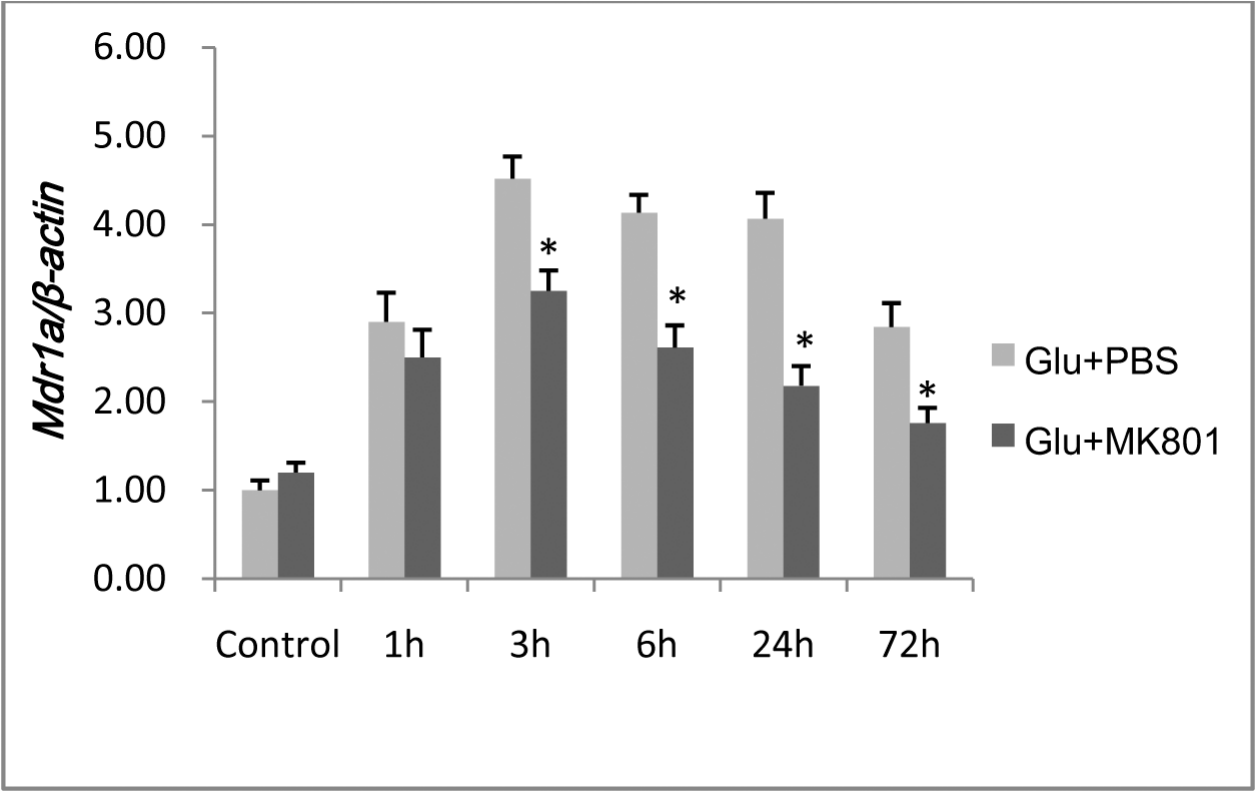
RT-PCR analysis of *mdr1a* gene expression in RBMECs. After L-glutamate stimulation, increased expression of *mdr1a* mRNA was detected in the Glu + PBS group. This effect could be attenuated after administration of MK-801 at 3, 6, 24, and 72 h. The asterisk represents *P*<0.05 between the Glu + PBS and MK801 + Glu groups.

RT-PCR analysis was used to investigate whether *mdr1a* mRNA expression was altered by L-glutamate in primary RBMECs. After a 1, 3, 6, 24, or 72 h incubation following exposure to 100 μM glutamate for 30 min, increased expression of *mdr1a* mRNA was detected. This increase could be prevented by MK-801(100 μM; Fig 5). These results suggested that up-regulation of *mdr1a* mRNA expression induced by L-glutamate is related to the excitation of NMDA receptors.

#### P-glycoprotein expression increased in L-glutamate-stimulated RBMECs, and was inhibited by MK-801

**Fig 6:**
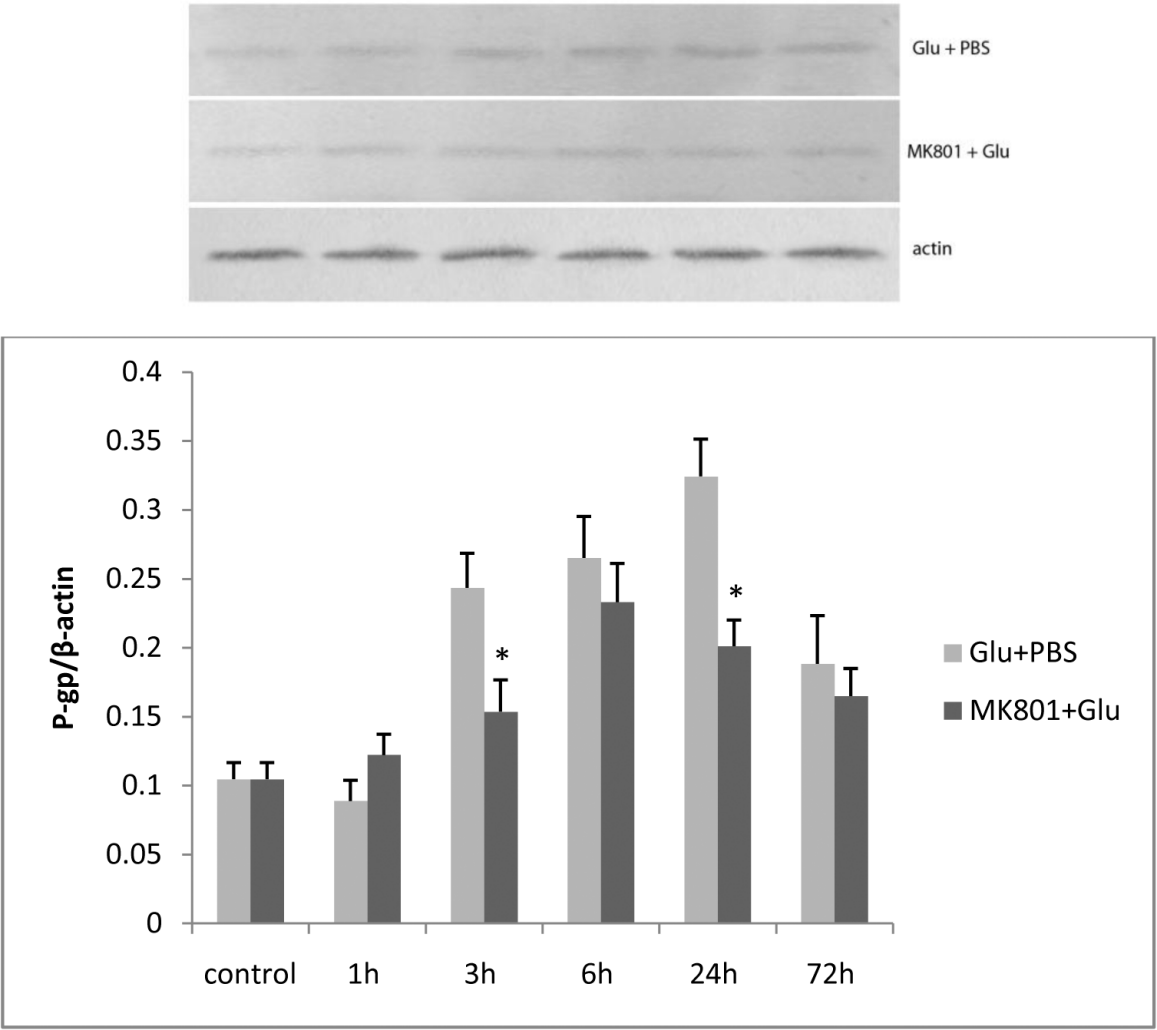
P-gp expression in L-glutamate stimulated RBMECs. After L-glutamate stimulation, increased expression of P-gp was detected in the Glu + PBS group at 3, 6, 24, and 72 h. This effect could be attenuated by pretreatment with MK-801 at 3, 6, 24, and 72 h. The asterisk represents *P*<0.05 between the Glu + PBS and MK-801 + Glu groups.

Next, we examined whether P-gp expression was influenced by L-glutamate. Increases in P-gp expression were found after 3, 6, 24, and 72 h incubations following treatment with 100 μM L-glutamate for 30 min. This effect of L-glutamate was attenuated by pretreatment with 100 μM MK-801 (Fig 6), suggesting that glutamate may play an important role in the up-regulation of P-gp expression.

#### eEF-2K expression increased in L-glutamate stimulated RBMECs

**Fig 7:**
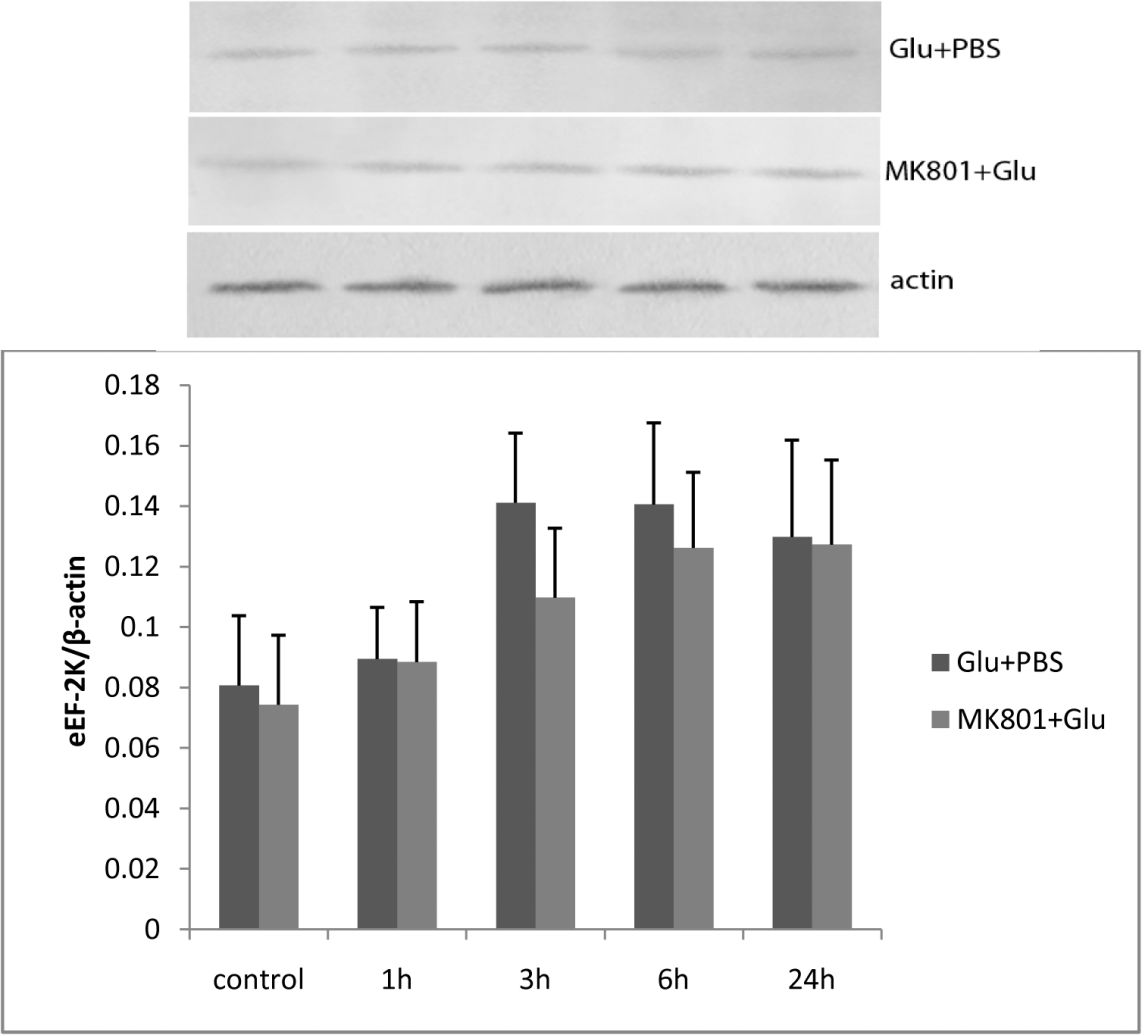
There was no significant change of eEF-2K expression found at any time points following treatment with 100 μM L-glutamate for 30 min. It had a tendency that eEF-2K expressed more at 3, 6, 24 h. eEF-2K expressed was tend to be attenuated by pretreatment with 100 μM MK-801.

We also investigated whether eEF-2K expression was influenced by L-glutamate. Our result showed no significant change of eEF-2K expression was found at any time points following treatment with 100 μM L-glutamate for 30 min. It showed a tendency that eEF-2K expressed more at 3, 6, 24 h. The effect of eEF-2K expressed was tend to be attenuated by pretreatment with 100 μM MK-801 (Fig 7).

#### Phospho-eEF-2 expression increased in L-glutamate stimulated RBMECs

**Fig 8:**
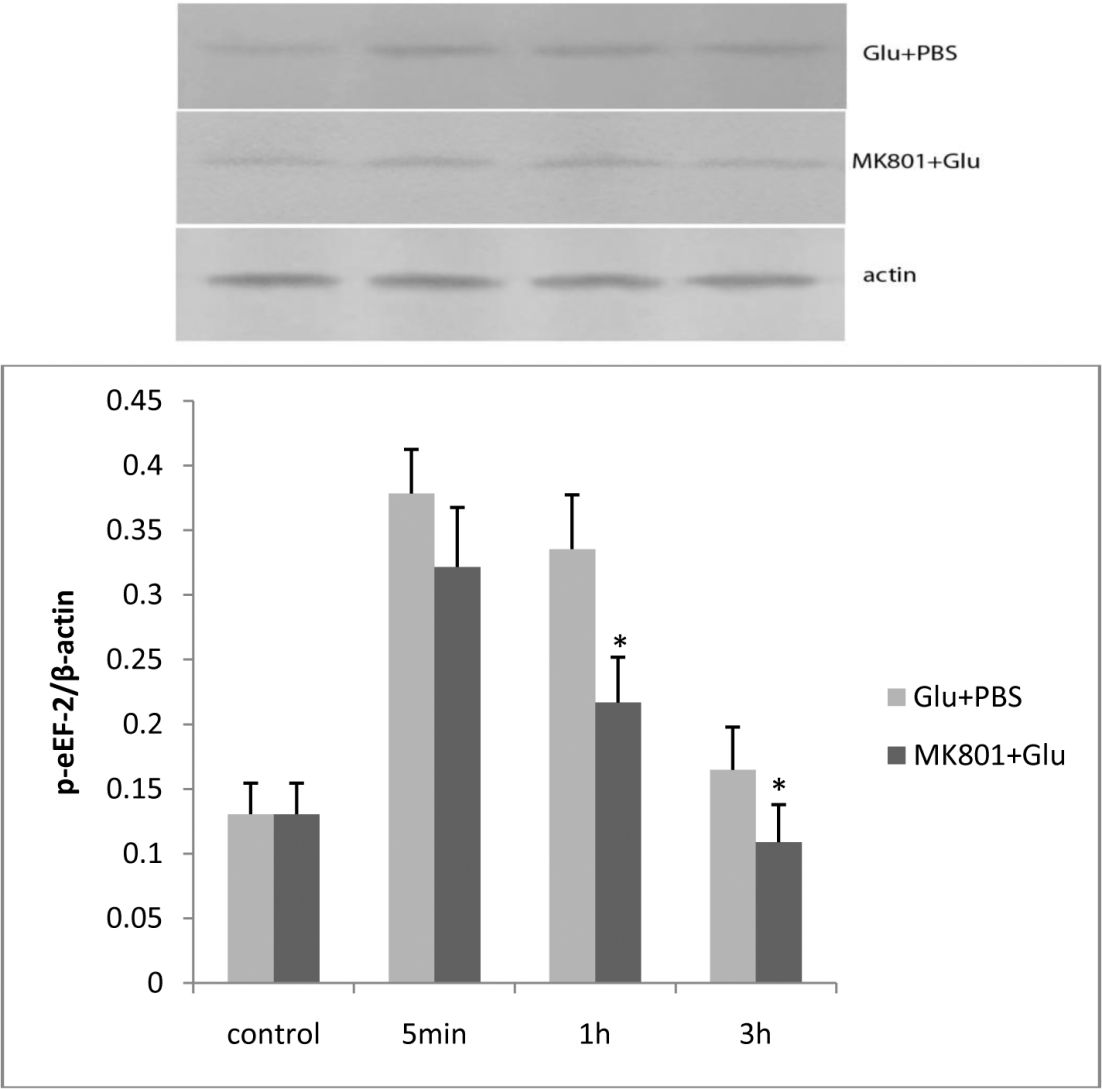
After L-glutamate stimulation, increased expression of phospho-eEF-2 was detected in the Glu + PBS group at 5 min, 1 h, and 3 h. The increase of phospho-eEF-2 expression was attenuated by MK-801. The asterisk represents *P*<0.05.

Next step, we investigated whether phospho-eEF-2 expression was influenced by L-glutamate. An increase of phospho-eEF-2 expression was found after a 5min and 1h incubation following treatment with 100 μM L-glutamate for 30 min. This effect of L-glutamate was attenuated by pretreatment with 100 μM MK-801 (Fig 8).

### Effect of NH125 stimulation on P-gp/phospho-eEF-2 expression

#### P-glycoprotein expression was down-regulated after NH125 treatment in L-glutamate stimulated RBMECs

**Fig 9:**
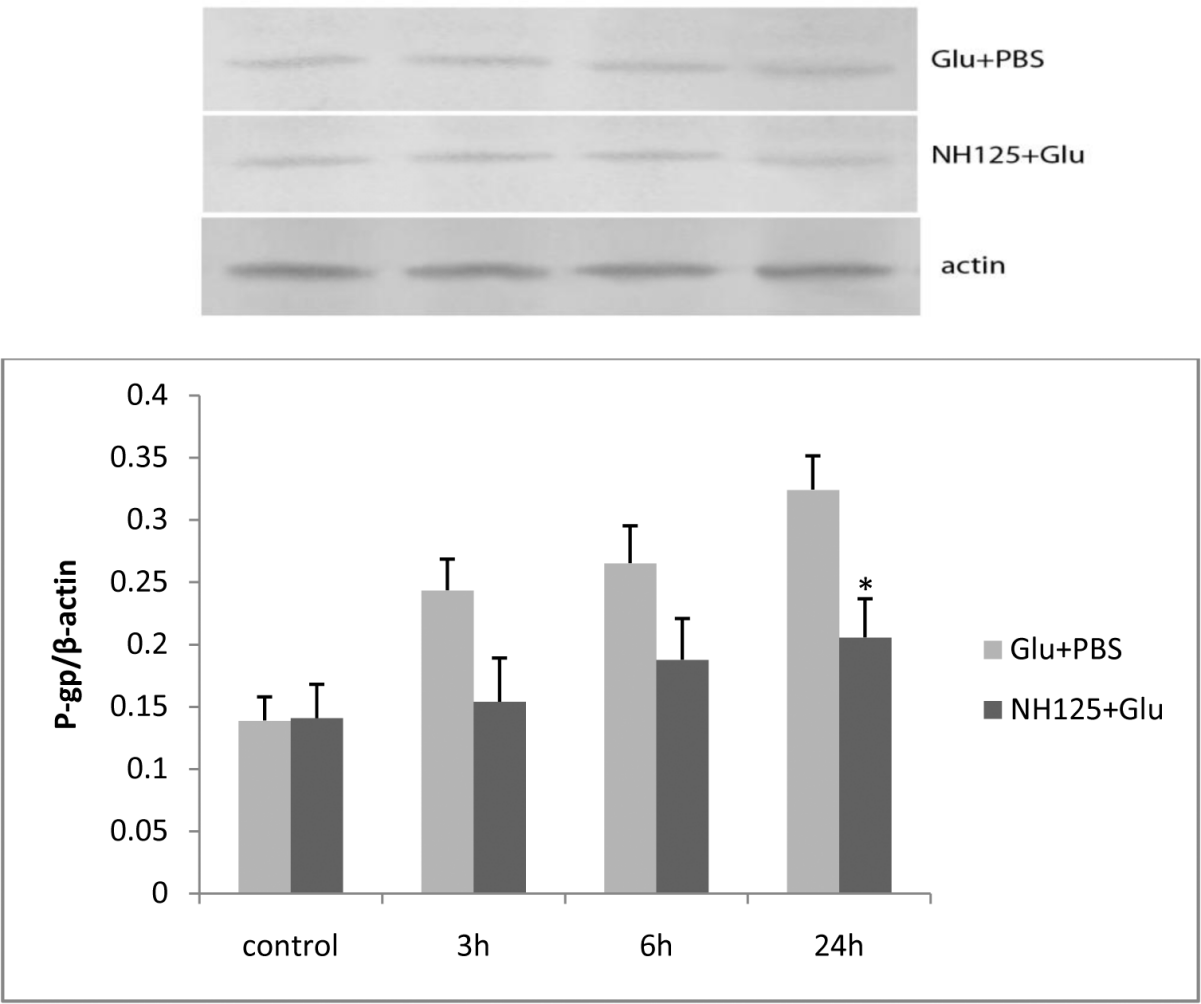
After adding 1 μM NH125, expression of P-gp was lower in the NH125 + Glu group than that of the Glu + PBS group at 3, 6, and 24 h. The asterisk represents *P*<0.05.

Our next goal was to investigate whether P-gp expression was influenced by NH125. A decrease of P-gp expression was found after 3, 6, and 24 h incubations following treatment with 1 μM NH125 in glutamate stimulated RBMECs. P-gp expression was significantly decreased in the NH125 + Glu group compared with that of the Glu + PBS group (Fig 9).

#### The expression of phospho-eEF-2 decreased after NH125 treatment in L-glutamate stimulated RBMECs

**Fig 10:**
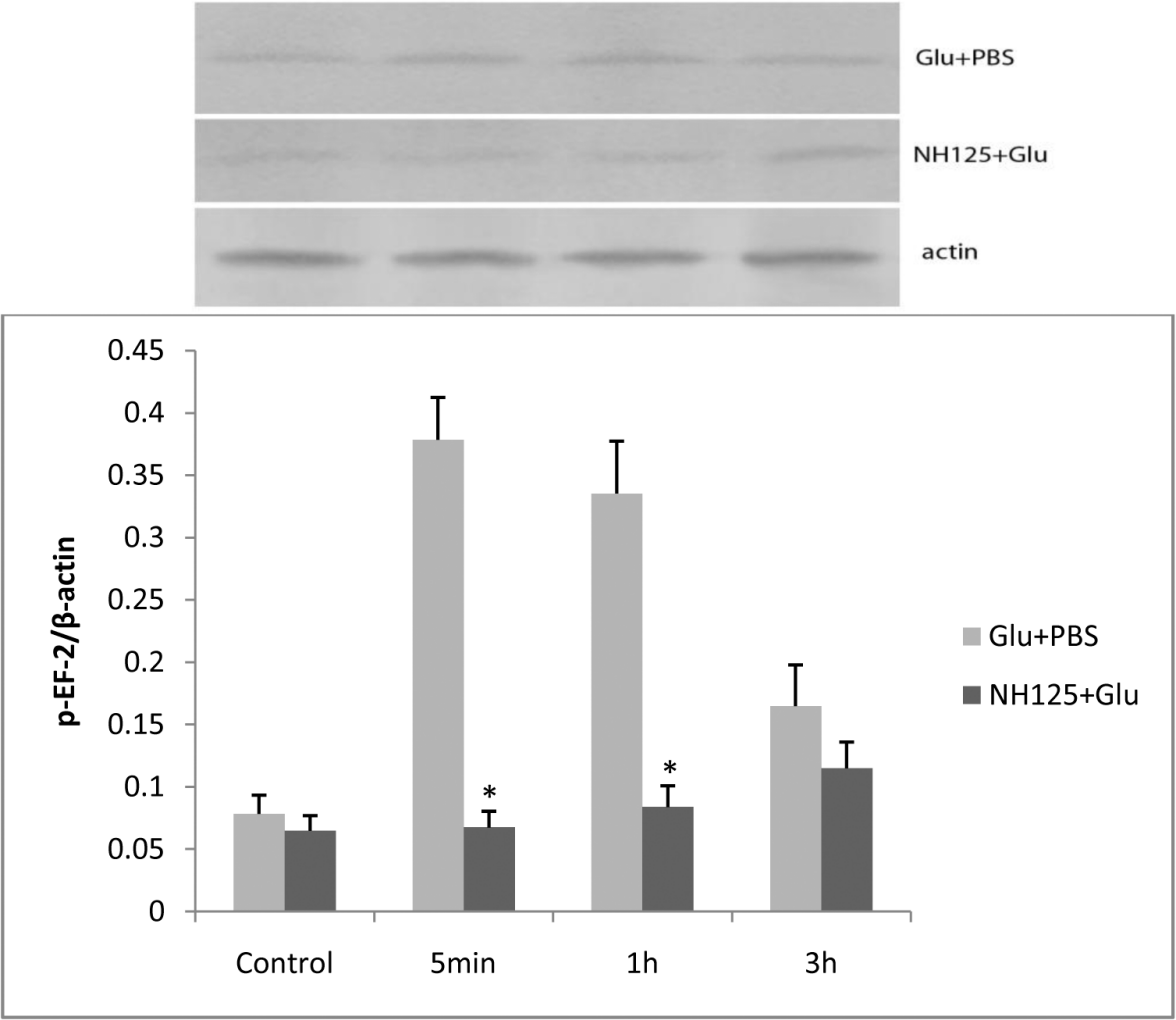
After adding NH125, expression of phospho-eEF-2 was lower in the NH125 + Glu group than in the Glu + PBS group at 5 min and 1 h. The asterisk represents *P*<0.05.

Our final goal was to determine whether phospho-eEF-2 expression was influenced by NH125. A decrease of phospho-eEF-2 expression was found after 5 min and 1 h incubations following treatment with 1 μM NH125 in glutamate stimulated RBMECs. Phospho-eEF-2 expression was significantly decreased in the NH125 + Glu group compared with that of the Glu + PBS group after 5 min and 1 h (Fig 10).

## Discussion

**Fig 11:**
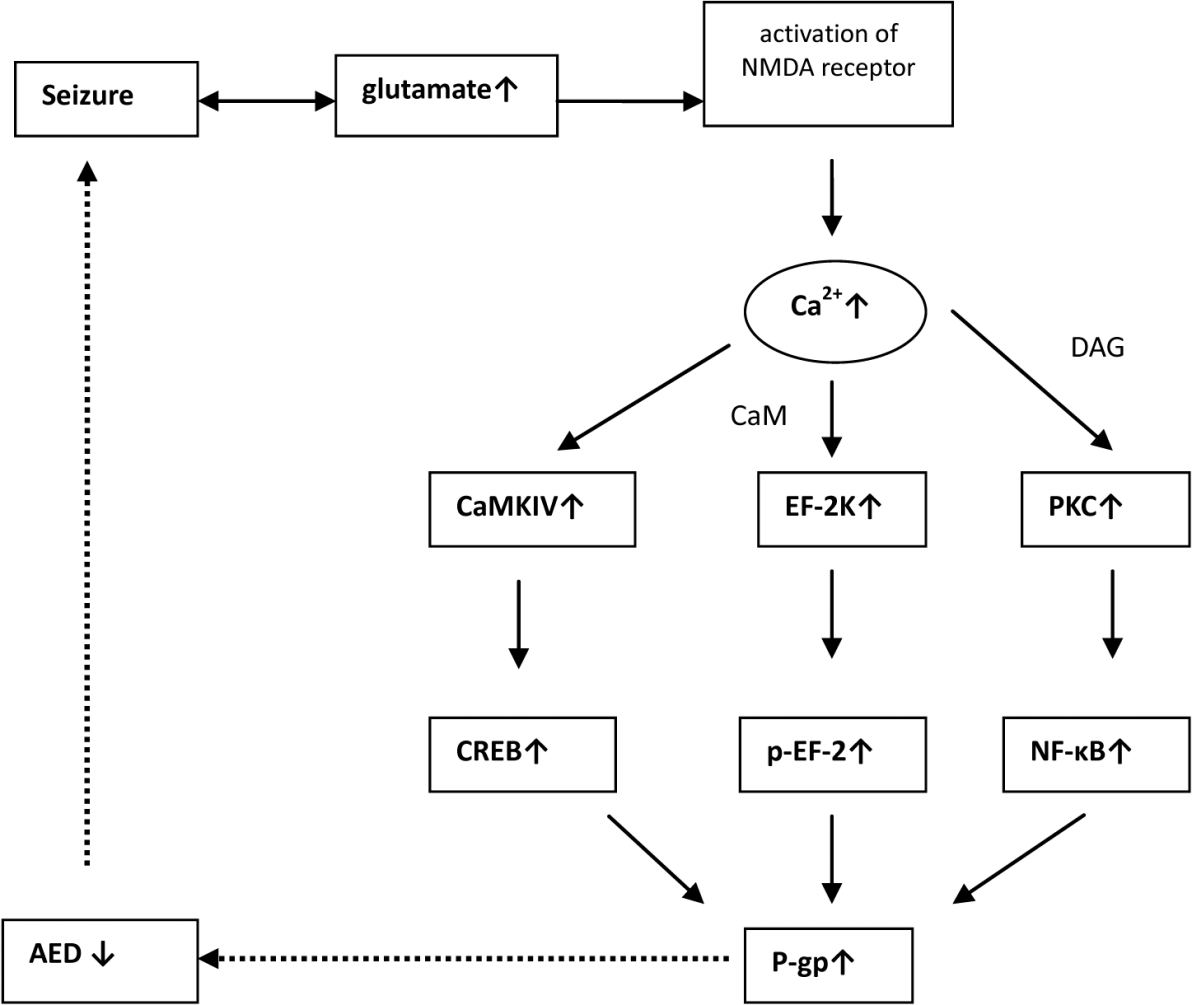
The whole schematic of proposed glutamate-NMDA receptor-P-gp signaling pathways. The other two signal pathways have been reported via increasing P-gp expression and turn to be drug resistant in several kind of caners.

This study was undertaken to investigate the possible pathway for glutamate up-regulating P-glycoprotein expression in RBMECs, which comprise the BBB, and the possible effect of the eEF-2K inhibitor NH125 on P-gp expression. In this study, we first confirmed the existence of the L-glutamate-NMDA receptor-Ca^2+^/calmodulin-eEF-2K/eEF-2-P-gp signaling pathway, and found that the NMDA receptor antagonist (MK-801) and eEF-2K inhibitor (NH125) inhibit P-gp over-expression in RBMECs (Fig 11).

Activation of glutamate receptors has been shown to mediate a large number of neuronal processes such as ischemia, anoxic damage, neuronal death, tumors[27–29] and epilepsy[1]. Studies have suggested that glutamate receptors play an important role in the function of the BBB. The BBB is necessary to provide an optimal chemical environment for cerebral function. Several layers exist between blood and the brain: capillary endothelial cells, a basement membrane that completely covers the capillaries, and finally, astrocyte processes that enclose the basement membrane. Each of these layers could potentially restrict the movement of solutes[30]. The BBB had previously been viewed as a passive system. The various facilitative transporters were considered to play a role in the regulation of brain metabolism through their ability to limit access. Ca^2+^ ATPases are now known to be found in the BBB. It is therefore plausible that, within the BBB, activation of NMDA receptors via glutamate can then activate the secondary messenger Ca^2+^, and take part in a series of neuronal processes.

An association between seizure-induced P-glycoprotein over-expression and resistance to antiepileptic drugs has been suggested by numerous studies in rodent epilepsy models and in epileptic tissue of pharmacoresistant patients. Recent studies also show that specific P-glycoprotein inhibitors can be used to overcome resistance in animal models of epilepsy. In our study, increased P-gp expression was induced by 100 μM glutamate, an effect that was attenuated by the NMDA receptor inhibitor MK-801. After RBMECs were stimulated with glutamate, we found that glutamate was involved in the regulation of P-gp expression, which was consistent with the P-gp expression found in vivo. Unfortunately, MK-801 did not completely prevent the up-regulation of P-gp. So there could also exist other signal molecules taking participate in regulating P-gp expression, or that MK-801 wasn't a complete competitive inhibitor of NMDA receptor.These results still suggested that activation of NMDA receptors may play an important role in the regulation of P-gp expression in RBMECs.

Our result only showed a tendency of eEF-2K expression increased after glutamate stimulation, but no significant change. We also found that phospho-eEF-2 expression increased after glutamate stimulation at 5 min, and 1 h in RBMECs, which could be attenuated by MK-801. This result was consistent with that of a previous study[10]. The eEF-2 kinase is a polypeptide of about 95–103 kDa, and activity of this purified kinase against eEF-2 is strictly dependent upon Ca^2+^ ions and calmodulin. eEF-2 kinase undergoes extensive auto-phosphorylation, which allows it to phosphorylate eEF-2 in the absence of added Ca^2+^ ions and calmodulin. The extreme C-terminus of eEF-2 kinase contains a key site for interaction with eEF-2[31]. Several studies have shown that an increasing level of phospho-eEF-2 was associated with inhibition of protein synthesis [32–35]. Park [36] found that most protein synthesis was inhibited in a mouse model of fragile X syndrome when eEF-2 was phosphorylated, while the expression of Arc/Arg 3.1 increased significantly. Marin [10] found that after neurons were stimulated by glutamate to induce NMDA receptor activation, eEF-2 was phosphorylated, and some protein expression was suppressed temporarily, while the expression of other related proteins was increased. For example, the transcription factor activator protein 1 (the activator protein 1, AP-1) family members, such as mRNA expression of *c-fos* and *c-jun*, were increased, which could induce expression of related proteins [9]. Therefore, we speculated that phospho-eEF-2 could activate related transcription factors that could increase related protein synthesis, while inhibiting protein synthesis. Such a mechanism would protect the body under a state of stress, promote early gene expression, and reinforce synthesis of the efflux protein P-gp, which could promote the excretion of excitatory toxic substances and other metabolites. It might play an important role in the body’s defense system and react to its physiological function.

The eEF-2K inhibitor NH125 can decrease protein synthesis, and this effect might be associated with changes in the phosphorylation status of several key components of the translational machinery[20,37]. At present, however, little is known about the signaling mechanisms regulating this process. In this study, NH125decreased the phosphorylation of the elongation factor eEF-2 (at Thr56), and the change coincided with a reduced activity of this protein. These findings initially appeared to be consistent because a decrease in eEF-2K activity was expected to account for the decrease in eEF-2 phosphorylation. Our results showed that inhibition of P-gp expression in RBMECs by NH125 is correlated with a decrease of eEF-2 phosphorylation, which partly confirmed our hypothesis. Additionally, data from the study raise a possibility that NH125-decreased eEF-2 phosphorylation may serve as a valuable marker to explore a novel mechanism for drug-resistant therapies.

Our preliminary data here suggest that NH125-induced eEF-2 phosphorylation is mediated through multiple pathways. At least two pathways, the AMPK and eEF-2K pathways, contribute to the increase of phospho-eEF-2. It remains unclear which other pathways are involved [24,37]. eEF-2K is a Ca^2+^/calmodulin-dependent enzyme. It has been reported that inhibition of eEF-2K by NH125 weakens as concentrations of calmodulin increase[37]. The results suggested that NH125 might compete with calmodulin. Calmodulin is important not only in eEF-2K but also in other cellular signaling pathways. If NH125 interferes with other calmodulin-dependent pathways, RBMECs treated with NH125 may produce unexpected patterns of eEF-2 phosphorylation. NH125 appears to be a potent anti-drug-resistant agent known to induce eEF-2 phosphorylation through regulation of multiple pathways.

There still were limitations in our study. It has clearly demonstrated that acute seizures induced in vivo by systemic administration of low doses of Kainate receptors, as well as excitotoxic cell death depend on GluK2-containing kainate receptors (KARs), whereas higher doses may also implicate activation of α-amino-3-hydroxy-5-methyl-4-isoxazole-propionic acid receptors (AMPARs). In our study, we focused on the influence of NMDAR on P-gp/eEF-2 expression, but we haven'tdone experiment onKAR or AMPAR. Therefore, we need further research about the effect of KAR/AMPAR on eEF-2/ P-gp when our result was applied in vivo.

The existence of the L-glutamate-NMDA receptor-eEF-2K/eEF-2 P-gp signaling pathway in RBMECs also needs further research in vivo. Phosphorylation of eEF-2, therefore, can serve as an indicator not only for identification of the remaining NH125 pathways, but also for the discovery of efficacious NH125-like anti-multidrug agents. Furthermore, the NMDA receptor antagonist MK-801 combined with the eEF-2K inhibitor NH125 might reinforce the effect of inhibiting P-gp over-expression in RBMECs.
